# Supplementary material for: Single cell analysis reveals distinct immune landscapes in transplant and primary sarcomas that determine response or resistance to immunotherapy
Source: Nat Commun. 2020 Dec 17;11:6410. doi: 10.1038/s41467-020-19917-0 (PMC7746723; doi:10.1038/s41467-020-19917-0)
Supplement: Supplementary file 1 — Supplementary Information [file 41467_2020_19917_MOESM1_ESM.pdf]

SUPPLEMENTARY INFORMATION

Supplementary Figure 1.

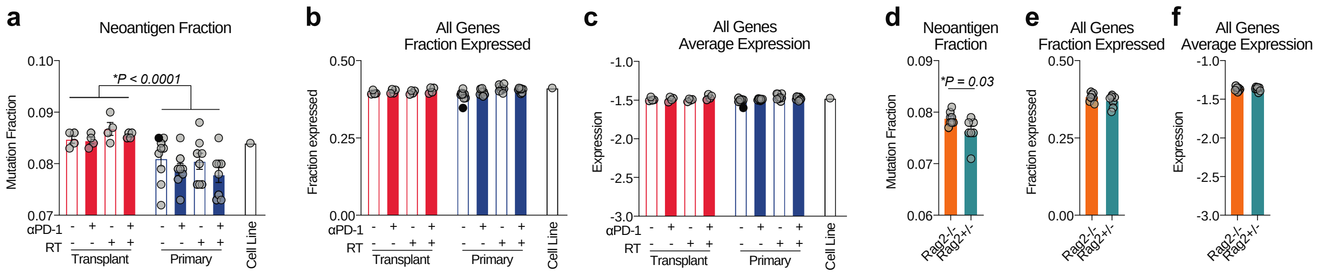

Supplementary Figure 1. Evidence of immune editing in primary tumors. a, Fraction of nonsynonymous mutations predicted to be neoantigenic in transplant and primary tumors harvested 3 days after specified treatment. The original primary tumor (filled, black) used to generate the cell line (right) from which the transplant tumors were derived is also shown. $P < 0.0001$ for transplant vs primary tumors. **b**, Fraction of all genes identified to be expressed (>0 FPKM) by RNA sequencing of transplant tumors, primary tumors, and the cell line. **c**, Average expression level of all genes in transplant tumors, primary tumors and the cell line quantified as fragments per kilobase of transcript per million mapped reads (FPKM), upper quartile normalized, $\log_2$ transformed. **d**, Fraction of nonsynonymous mutations in primary p53/MCA tumors from *Rag2*$^{-/-}$ and *Rag2*$^{+/-}$ mice predicted to be neoantigenic. **e**, Fraction of all genes identified to be expressed (>0 FPKM) by RNA sequencing of primary tumors from *Rag2*$^{-/-}$ and *Rag2*$^{+/-}$ mice. **f**, Average expression level of all genes in tumors from *Rag2*$^{-/-}$ and *Rag2*$^{+/-}$ mice, quantified as FPKM, upper quartile normalized, $\log_2$ transformed. For **a-f**, each symbol represents an individual mouse. Mean ± SEM. **a-c**, Transplant tumors: n = 4; primary tumors: n = 8. Significance determined by three-way ANOVA with Tukey's multiple comparisons test. **d-f**, *Rag2*$^{-/-}$ tumors: n = 8; *Rag2*$^{+/-}$ tumors: n = 7. Significance determined by unpaired two-tailed t-test. Source data are provided as a Source Data file.

Supplementary Figure 2.

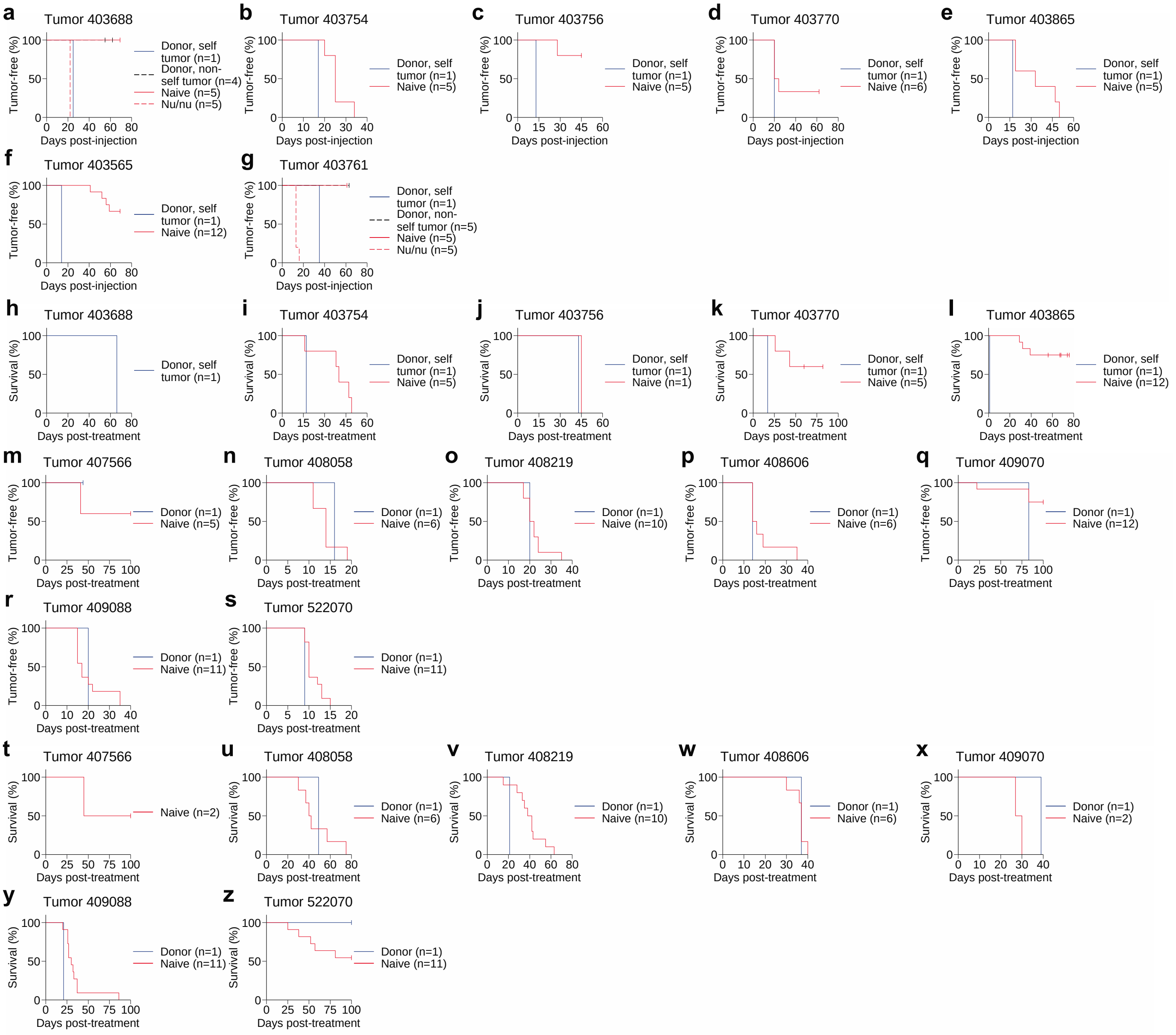

Supplementary Figure 2. Tumor outgrowth and survival after cell line injection into donor and naive mice. a-g, Time to tumor onset data from Fig. 3b and 3e, separated by cell line. *Nu/nu* (**a**, **g**) indicates cell line injection into a T-cell deficient athymic (nude) mice. **h-l**, Survival after treatment with 10 Gy + anti-PD-1 from Fig. 3c, separated by cell line. **m-s**, Time to tumor onset data from Fig. 3g, separated by cell line. **t-z**, Survival after treatment with 20 Gy + anti-PD-1 from Fig. 3h, separated by cell line. Survival curves estimated using Kaplan-Meier method. Source data are provided as a Source Data file.

Supplementary Figure 3.

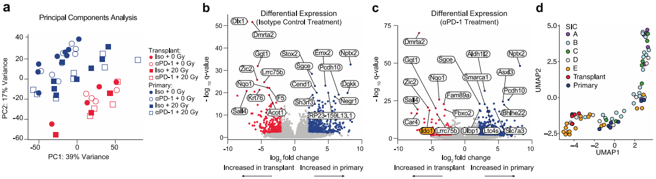

Supplementary Figure 3. Transcriptional analysis of primary and transplant sarcomas. a, Principal components analysis of bulk tumor RNA sequencing harvested 3 days after treatment. Each symbol represents an individual mouse's tumor. **b**, Volcano plot showing the log-fold-changes and Benjamini Hochberg-corrected p-values (q-values) for differential expression between transplant and primary tumors treated with isotype control antibodies. **c**, Volcano plot showing the log-fold-changes and Benjamini Hochberg-corrected p-values (q-values) for differential expression between transplant and primary tumors treated with anti-PD-1 antibodies. For **b** and **c**, genes with a negative log-fold-change are more highly expressed among transplant tumors (red), and genes with a positive log-fold change are more highly expressed in primary tumors (blue). Genes are colored blue or red if log-fold-change > 4 or < -4 and Benjamini-Hochberg adjusted p value < 0.05 (Wald test). **d**, Uniform manifold approximation and projection (UMAP) representation of cellular composition quantified by bulk RNA-seq followed by CIBERSORTx analysis of human and murine sarcomas. For the human sarcomas, undifferentiated pleomorphic sarcomas were analyzed and previously defined SIC is indicated. Source data are provided as a Source Data file.

3

Supplementary Figure 4.

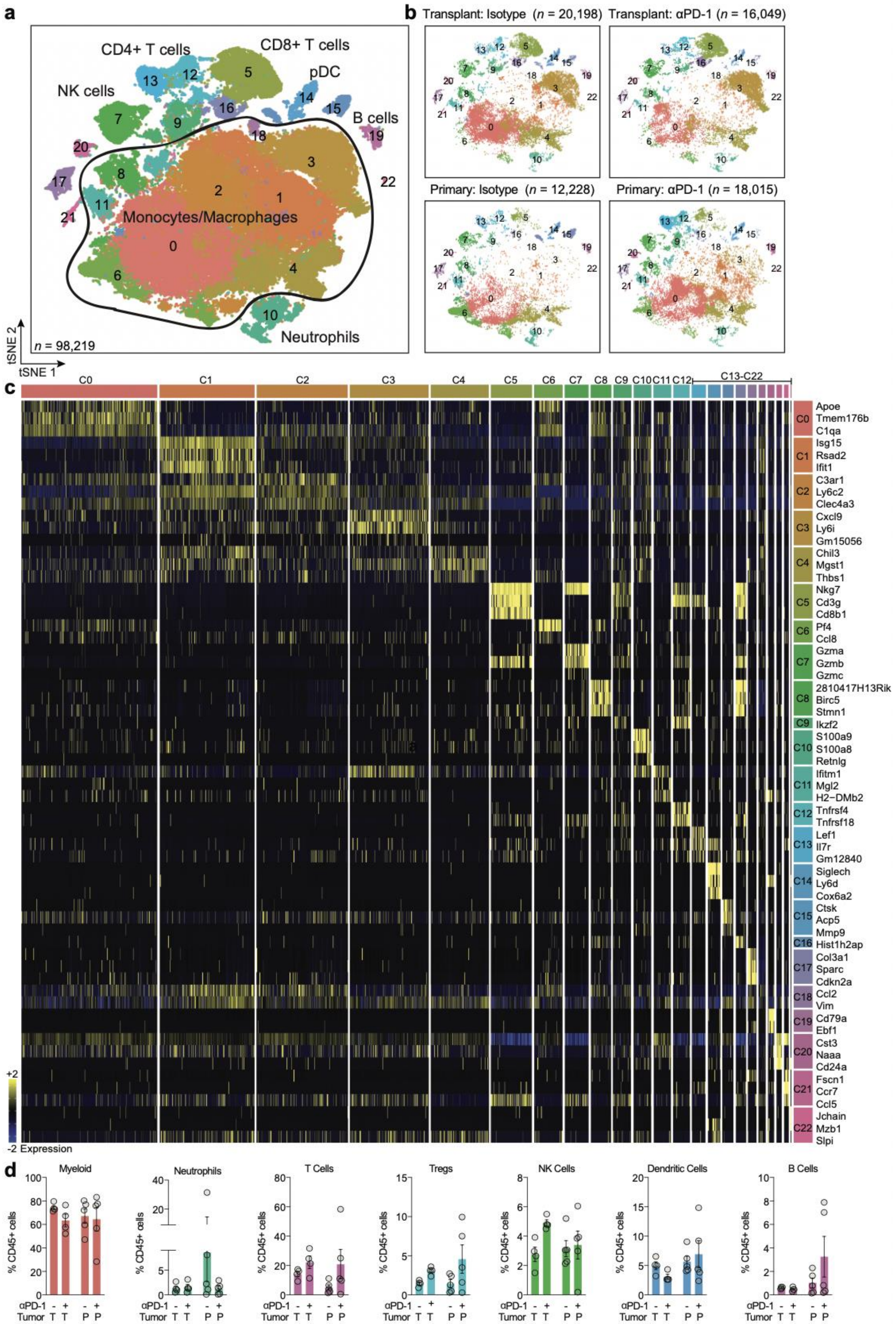

Supplementary Figure 4. Characterization of immune microenvironment by single-cell RNA sequencing. a, CD45+ immune cells from all tumor and treatment groups, overlaid on tSNE representation and color coded by cluster. Cell populations expressing genes consistent with monocyte/macrophage phenotype are outlined. **b**, Distribution of immune cells separated by indicated tumor and treatment type. **c**, Scaled expression values of top three discriminative genes per cluster. **d,** Bar graphs comparing frequency of indicated cell type (% CD45+) from transplant (T) or primary (P) tumors after αPD-1 (+) or isotype control (-). Each symbol represents an individual mouse; n = 4 per group for transplant tumors, n = 5 per group for primary tumors. Data show mean ± SEM. Source data are provided as a Source Data file.

Supplementary Figure 5.

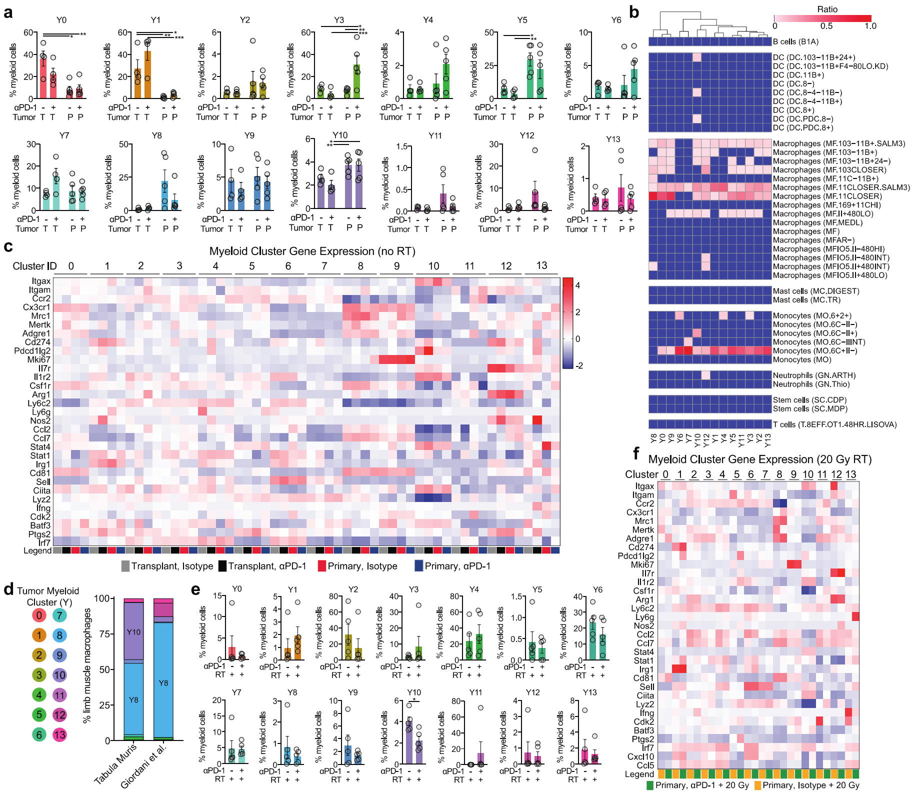

Supplementary Fig. 5. Defining myeloid populations using scRNA-seq. a, Bar graphs comparing frequency of indicated cell type (% myeloid cells) from transplant (T) or primary (P) tumors after αPD-1 (+) or isotype control (-). Data show mean ± SEM, **P <0.05* by 2-way ANOVA and Tukey's multiple comparisons test. Each symbol represents an individual mouse; n = 4 per group for transplant tumors, n = 5 per group for primary tumors. Y0: **P*=0.0032, ***P*=0.0043; Y1: **P* =0.0226, ***P*=0.012, ****P*=0004; Y3: **P* =0.028, ***P*=0.011, ****P*=0.0069; Y5: **P* =0.037, ***P*=0.0129; Y10: **P* =0.0497 , ***P*=0.0478. **b**, SingleR analysis of myeloid clusters, annotated based on correlation profiles with bulk RNA-seq from the Immunological Genome Project

6

(ImmGen) database. The heatmap color scale corresponds to the ratio of cells attributed to that cell type for each cluster. **c**, Heat map comparing expression of genes in indicated clusters and treatment groups. **d**, Single-cell RNA-seq data from normal limb muscle macrophages published by the Tabula Muris Consortium[1] and Giordani *et al.*[2] compared to tumor myeloid clusters Y0-Y14. The tumor myeloid cluster most similar to each normal muscle macrophage cell was identified, and the percent of normal limb muscle macrophage cells most similar to each myeloid cluster is reported. **e**, Bar graphs comparing frequency of indicated cell type (% myeloid cells) from primary tumors 3 days after 20 Gy RT and either αPD-1 (+) or isotype control (-). Data show mean ± SEM, Y10: *P=0.0145 by two-tailed unpaired t-test. Each symbol represents an individual mouse; n = 5 per group. **f**, Heat map comparing expression of genes in indicated clusters. Source data are provided as a Source Data file.

Supplementary Figure 6.

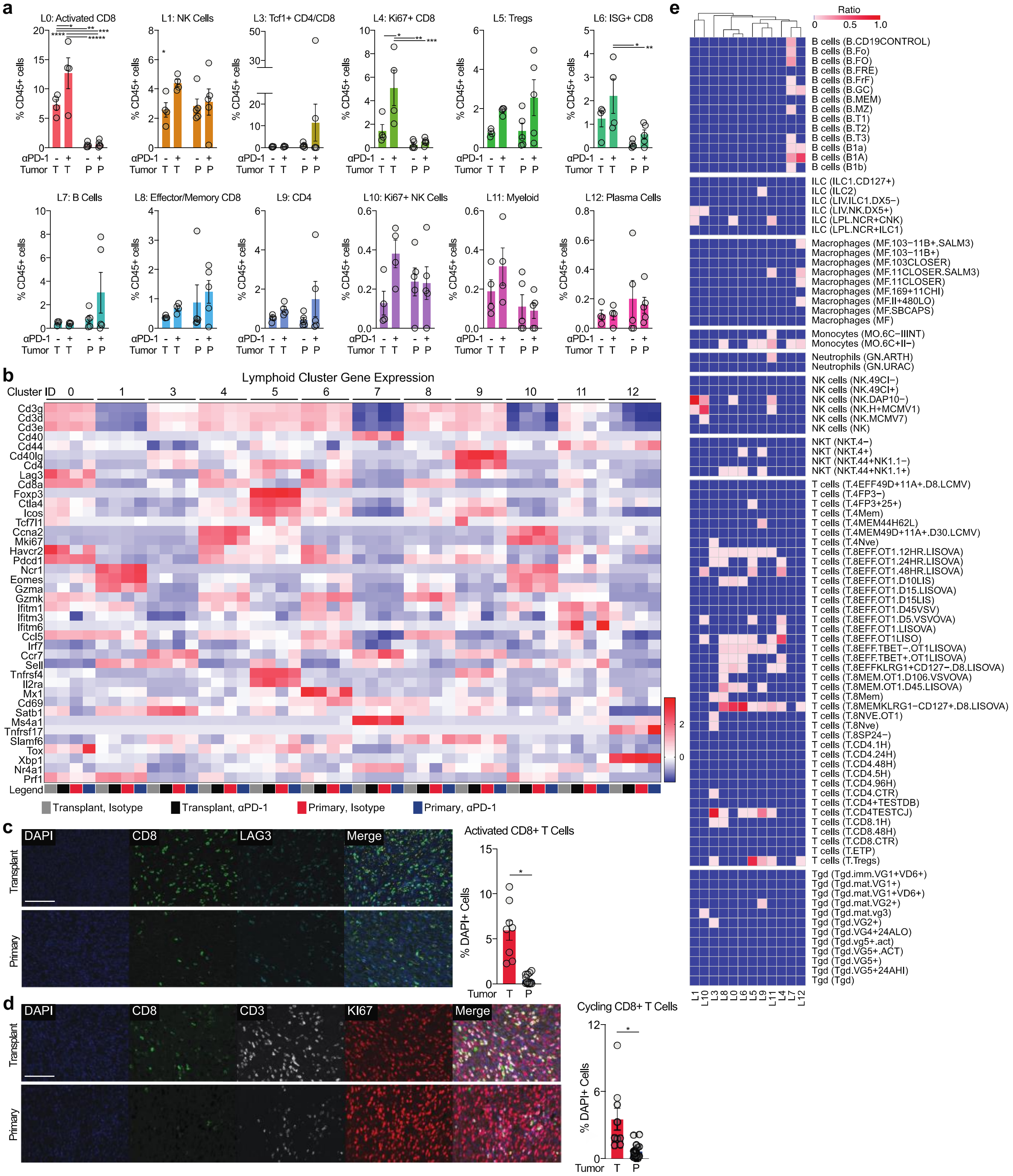

Supplementary Fig. 6. Phenotypes of lymphoid populations. a, Bar graphs comparing frequency of indicated cell type (% lymphoid cells) from transplant (T) or primary (P) tumors after αPD-1 (+) or isotype

control (-). Each symbol represents an individual mouse; n = 4 per group for transplant tumors, n = 5 per group for primary tumors. Data show mean ± SEM. *$P < 0.05$* by 2-way ANOVA and Tukey's multiple comparisons test. L0: *P=0.0478, **P=0.007, ***P=0.0083, ****P<0.0001, *****P<0.0001; L4: *P=0.0189, **P=0.0015, ***P=0.0024; L6: *P=0.0081, **P=0.0376. **b**, Heat map comparing expression of genes in indicated clusters and treatment groups. **c**, Immunofluorescence staining for activated CD8+ T cells in transplant (T, top, n = 8) or primary (P, bottom, n = 16) tumors harvested 3 days after αPD-1 treatment. All images were taken at the same magnification, so the scale bar in the top left image applies to all images in panel **c**. Scale bar = 100 µm. Quantification of images (right), *P<0.0001 by two-tailed unpaired t-test. Data show mean ± SEM. **d**, Immunofluorescence staining for cycling CD8+ T cells (Ki67+) in transplant (T, top, n = 9) or primary (P, bottom, n = 19) tumors harvested 3 days after αPD-1 treatment. All images were taken at the same magnification, so the scale bar in the top left image applies to all images in panel **d**. Scale bar = 100 µm. Quantification of images (right), *P=0.0003 by two-tailed unpaired t-test. Data show mean ± SEM**. e**, SingleR analysis of lymphoid clusters. The heatmap color scale corresponds to the ratio of cells attributed to that cell type for each cluster. Source data are provided as a Source Data file. For **c** and **d**, three tissue slices from each independent tumor were stained, quantified, and the resulting 3 values were averaged together to obtain a single value from each tumor. Sample sizes reflect the number of independent tumors (each from a separate mouse) that were used for staining. Each dot represents the average quantification from 3 tissue sections from a single tumor.

Supplementary Figure 7.

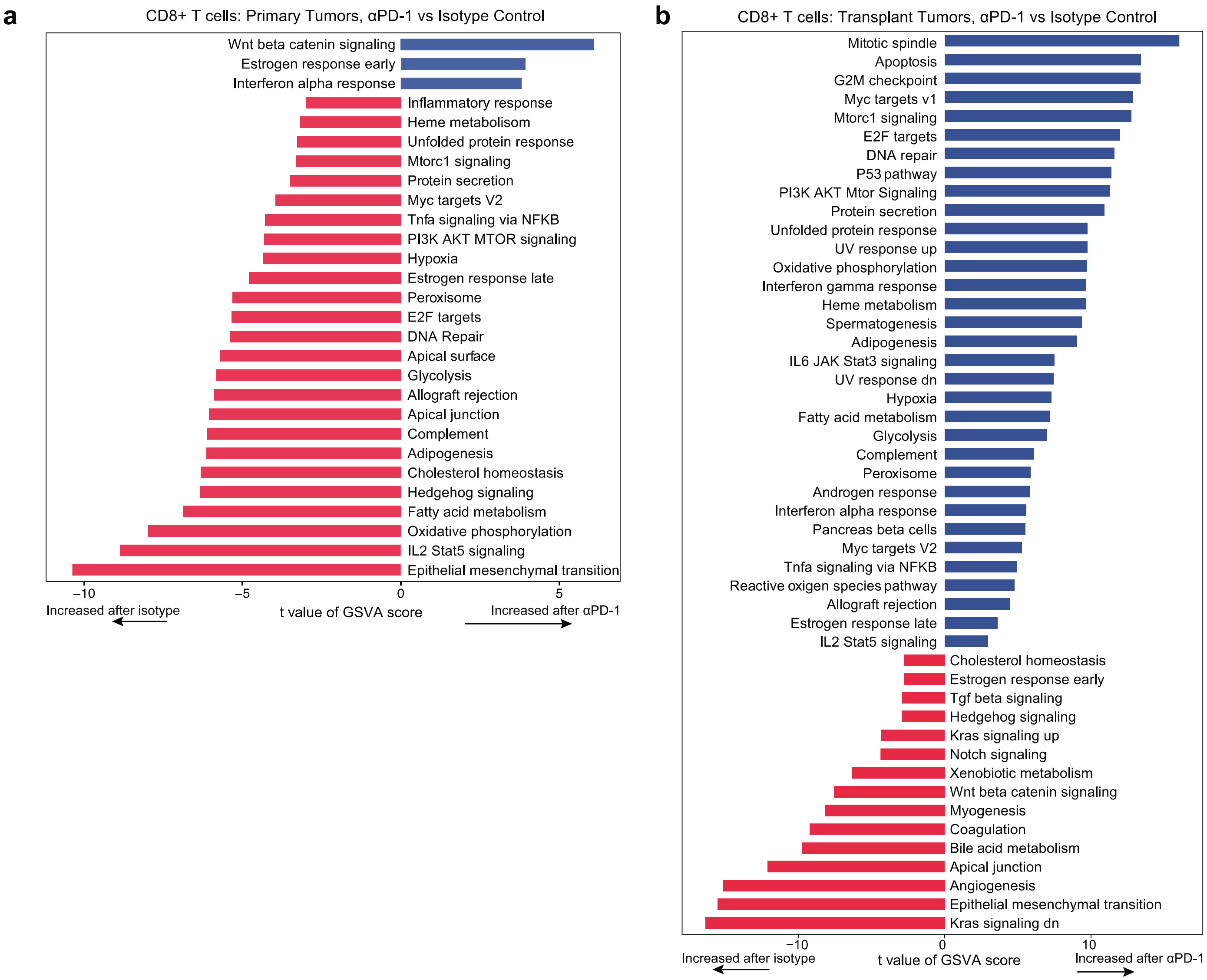

Supplementary Figure 7. Transcriptional signatures of CD8+ T cells. a, Pathways with significantly different activities scored per cell comparing gene set variation analysis (GSVA) enrichment scores between CD8+ T cells from primary tumors treated with αPD-1 vs isotype control antibody (n = 2,473 cells analyzed). **b**, Pathways with significantly different activities scored per cell comparing GSVA enrichment scores between CD8+ T cells from transplant tumors treated with αPD-1 vs isotype control antibody (n = 5,828 cells analyzed). Plotted are t statistics from a simple linear model. Benjamini-Hochberg method was used to adjust p values for multiple hypothesis testing. Pathways shown are significant at false discovery rate <0.01.

SUPPLEMENTARY TABLE 1

Reagent/Resource	Source	Catalogue Number	Dilution
Anti-mouse CD45 (Clone 30-F11) BV605	Biolegend	103139	1:100
Anti-mouse CD45 (Clone 30-F11) APC-Cy7	Biolegend	103116	1:100
Anti-Mouse CD11b (M1/70)-148Nd	Fluidigm	3148003B	1:400
Anti-Mouse CD19 (6D5)-166Er	Fluidigm	3166015B	1:200
Anti-Mouse CD45 89Y	Fluidigm	3089005B	1:200
Anti-Mouse CX3CR1 (SA011F11)-164Dy A18	Fluidigm	3164023B	1:200
Anti-Mouse CD11c (N418)-142Nd	Fluidigm	3142003B	1:100
Anti-Human/Mouse CD44 (IM7)-171Yb	Fluidigm	3171003B	1:100
Anti-Mouse I-A/IE(M5/114.15.2)-209Bi	Fluidigm	3209006B	1:100
Anti-mouse CD335 (NKp46)	Biolegend	29A1.4	1:100
Anti-Mouse CD154/CD40 Ligand (MR1)-170Er	Fluidigm	3170011B	1:100
Anti-Mouse Ly-108 Monoclonal Antibody (13G3-19D)	ThermoFisher	14-1508-82	1:100
Anti-Mouse F4/80 (BM8)-146Nd	Fluidigm	3146008B	1:50
Anti-Mouse CD366/TIM-3 (RMT3-23)-162Dy	Fluidigm	3162029B	1:50
Anti-Mouse CD278 ICOS (7E.17G9) 176Yb	Fluidigm	3176014B	1:50
Anti-Mouse CD4 (RM4-5)-145Nd	Fluidigm	3145002B	1:50
Anti-Mouse CD62L(MEL-14)-160Gd	Fluidigm	3160008B	1:50
Anti-Mouse CD8a (53-6.7)-168Er	Fluidigm	3168003B	1:50
Anti-Mouse Ly-6C (HK1.4)-150Nd	Fluidigm	3150010B	1:50
Anti-Mouse Ly-6G (1A8)-141Pr	Fluidigm	3141008B	1:50
Anti-Mouse MHC Class I (28-14-8)- 144Nd	Fluidigm	3144016B	1:50
Anti-Mouse CD274/PD-L1 (10F.9G2)-153Eu	Fluidigm	3153016B	1:50
Anti-mouse CD124 (I015F8)	Biolegend	144802	1:50
Anti-mouse Siglec H antibody (Clone 551)	Biolegend	129602	1:50
Anti-mouse CD103 Antibody (Clone 2E7)	Biolegend	121402	1:50
Anti-mouse CD223/Lag3 Antibody (Clone C9B7W)	Biolegend	125202	1:50
Anti-Mouse CCR2	R&D Systems	MAB55381-100	1:50
Anti-Mouse CD3e (145-2C11)-152Sm	Fluidigm	3152004B	1:25
Anti-Mouse/Rat CD40 (HM40-3)-161Dy	Fluidigm	3161020B	1:25
Anti-Mouse CD206/MMR (C068C2)-169Tm	Fluidigm	3169021B	1:100
Anti-mouse Nos2 (clone 5C1B52)	Biolegend	690902	1:100
Anti-Mouse Bcl2 (10C4)	ThermoFisher	14-6992-82	1:100
Anti-Mouse CD152 antibody (Clone 9H10)	Biolegend	106202	1:100
Anti-mouse EOMES (clone dan11mag)	ThermoFisher	14-4875-82	1:67

SUPPLEMENTARY TABLE 1, CONTINUED

Anti-Mouse CD279/PD-1 (29F.1A12)-159Tb	Fluidigm	3159024B	1:50
Anti-Human Ki-67(B56)-172Yb	Fluidigm	3172024B	1:50
Anti-Mouse Foxp3(FJK-16s)-158Gd	Fluidigm	3158003A	1:50
Anti-Human Granzyme B (GB11)-173Yb	Fluidigm	3173006B	1:25
TCF1/TCF7 (C63D9) Rabbit mAb	Cell Signaling	2203S	1:16
Maxpar X8 Antibody Labeling Kit, 155Gd	Fluidigm	201155A	n/a
Maxpar X8 Antibody Labeling Kit, 174Yb	Fluidigm	201174A	n/a
Maxpar X8 Antibody Labeling Kit, 156Gd	Fluidigm	201156A	n/a
Maxpar X8 Antibody Labeling Kit, 147Sm	Fluidigm	201147A	n/a
Maxpar X8 Antibody Labeling Kit, 143Nd	Fluidigm	201143A	n/a
Maxpar X8 Antibody Labeling Kit, 154Sm	Fluidigm	201154A	n/a
Maxpar X8 Antibody Labeling Kit, 165Ho	Fluidigm	201165A	n/a
Maxpar X8 Antibody Labeling Kit, 151Eu	Fluidigm	201151A	n/a
Maxpar X8 Antibody Labeling Kit, 167Er	Fluidigm	201167A	n/a
Maxpar X8 Antibody Labeling Kit, 149Sm	Fluidigm	201149A	n/a
Maxpar X8 Antibody Labeling Kit, 175Lu	Fluidigm	201175A	n/a
Foxp3 / Transcription Factor Staining Buffer Set	ThermoFisher	00-5523-00	n/a
Cell-ID 20-Plex Pd Barcoding Kit	Fluidigm	201060	n/a
Mouse Tumor Dissociation Kit	Miltenyi Biotec	130-096-730	n/a
Zombie Yellow Fixable Viability Kit	Biolegend	423103	1:400
Cell-ID Cisplatin 195	Fluidigm	201064	1:4000
Cell-ID Intercalator-Ir—125 µM	Fluidigm	201192A	1:2000
16% Formaldehyde Solution	Thermo Fisher	28906	n/a
RPMI 1640 Media	ThermoFisher	11875135	n/a
Fetal Bovine Serum, heat inactivated	ThermoFisher	16140071	n/a
Antibody Stabilizer	Candor	131 125	n/a
HBSS, no calcium, no magnesium, no phenol red	ThermoFisher	14175103	n/a

SUPPLEMENTARY TABLE 2

Immunoflourescence Staining Reagents							
Panel	Position	Antibody	Clone / Host	Company / Item	Concentration	Secondary	OPAL Fluor
1	1	CD3	CD3-12 / Rat	Bio-Rad / MCA1477	0.5ug/ml	Rat immpress	570
1	2	CD4	4SM95 / Rat	Invitrogen / 14-9766-82	2.5ug/ml	Rat immpress	690
1	3	CD8a	4SM15 / Rat	Invitrogen / 14-0808-82	0.5ug/ml	Rat immpress	520
1	4	FoxP3	FJK-16S / Rat	eBioscience / 14-5773-82	0.125ug/ml	Rat immpress	620
1	5	Ki67	D3B5 / Rabbit	Cell Signaling / 12202S	0.1ug/ml	Rabbit powervision	650
2	1	CD8a	4SM15 / Rat	eBioscience / 14-0808-82	0.5ug/ml	Rat immpress	480
2	2	LAG-3	EPR2029 4-77 / Rabbit	Abcam / ab209238	0.98ug/ml	Rabbit powervision	520
2	4	CD3	CD3-12 / Rat	Bio-Rad / MCA1477	3.34 ug/ml	Rat immpress	690
1, 2	n/a	Rat ImmPress HRP Polymer	secondary	Vector Labs / MP-7444-15	RTU	n/a	n/a
1, 2	n/a	Rabbit PowerVision HRP Polymer	secondary	Leica / PV6119	RTU	n/a	n/a

SUPPLEMENTARY REFERENCES

1. Tabula Muris Consortium *et al.* Single-cell transcriptomics of 20 mouse organs creates a Tabula Muris. *Nature* **562**, 367–372 (2018).
2. Giordani, L. *et al.* High-Dimensional Single-Cell Cartography Reveals Novel Skeletal Muscle-Resident Cell Populations. *Mol. Cell* **74**, 609–621.e6 (2019).
