# Supplementary material for: Single cell analysis reveals distinct immune landscapes in transplant and primary sarcomas that determine response or resistance to immunotherapy
Source: Nat Commun. 2020 Dec 17;11:6410. doi: 10.1038/s41467-020-19917-0 (PMC7746723; doi:10.1038/s41467-020-19917-0)
Supplement: Supplementary file 3 — Descriptions of Additional Supplementary Files [file 41467_2020_19917_MOESM3_ESM.pdf]

Descriptions of Additional Supplementary Files.

Supplementary Data 1

Description: CIBERSORTx analysis of TCGA sarcomas and murine tumors. Enumeration of cellular proportions by bulk tumor RNA-seq.

Supplementary Data 2

Description: scRNA-Seq differential gene expression in myeloid cells. a, Differentially expressed genes in CD14+ cells from primary tumors treated with anti-PD1 and 0 Gy vs transplant tumors treated with anti-PD-1 and 0 Gy. b, Differentially expressed genes in CD14+ cells from primary tumors treated with isotype control and 20 Gy vs primary tumors treated with isotype control and 0 Gy. c, Differentially expressed genes in CD14+ cells from primary tumors treated with anti-PD1 and 0 Gy vs primary tumors treated with isotype control and 0 Gy. d, Differentially expressed genes in CD14+ cells from primary tumors treated with anti-PD1 and 20 Gy vs primary tumors treated with isotype control and 20 Gy. e, Differentially expressed genes in CD14+ cells from transplant tumors treated with anti-PD1 and 0 Gy vs transplant tumors treated with isotype control and 0 Gy. f, Differentially expressed genes in CD14+ cells from primary tumors treated with isotype control and 0 Gy vs transplant tumors treated with isotype control and 0 Gy. g, Differentially expressed genes in CD14+ cells from primary tumors treated with anti-PD1 and 20 Gy vs primary tumors treated with anti-PD-1 and 0 Gy.

Supplementary Data 3

Description: scRNA-Seq cluster markers. Markers for clusters identified in all cells, in lymphoid clusters, and in myeloid clusters. a, Gene expression markers used to determine markers for each cluster on tSNE plot with all cells (Supplementary Figure 4a). b, Gene expression markers used to determine markers for each cluster on tSNE plots of myeloid cells (Figure 5c). c, Gene expression markers used to determine markers for each cluster on tSNE plots of lymphoid cells (Figure 6e).

Supplementary Data 4

Description: scRNA-Seq differential gene expression in CD8+ T cells. a, Differentially expressed genes in CD8+ cells from primary tumors treated with anti-PD1 and 0 Gy vs transplant tumors treated with anti-PD-1 and 0 Gy. b, Differentially expressed genes in CD8+ cells from primary tumors treated with isotype control and 20 Gy vs primary tumors treated with isotype control and 0 Gy. c, Differentially expressed genes in CD8+ cells from primary tumors treated with anti-PD1 and 0 Gy vs primary tumors treated with isotype control and 0 Gy. d, Differentially expressed genes in CD8+ cells from primary tumors treated with anti-PD1 and 20 Gy vs primary tumors treated with isotype control and 20 Gy. e, Differentially expressed genes in CD8+ cells from transplant tumors treated with anti-PD1 and Health Duke University Medical Center BOX DUMC 91006, Durham, NC 27710 LOC B330 LSRC Bldg. Durham, NC 27708 TEL 919-681-8605 FAX 919-681-1867 EMAIL david.kirsch@duke.edu URL www.kirschlab.org www.dukehealth.org 0 Gy vs transplant tumors treated with isotype control and 0 Gy. f, Differentially expressed genes in CD8+ cells from primary tumors treated with isotype control and 0 Gy vs transplant tumors treated with isotype control and 0 Gy. g, Differentially expressed genes in CD8+ cells from primary tumors treated with anti-PD1 and 20 Gy vs primary tumors treated with anti-PD-1 and 0 Gy.

Supplementary Data 5

Description: Bulk tumor RNA-seq summary. Summaries for alignment and mapping performance for primary and transplant bulk tumor RNA seq.

Supplementary Data 6

Description: Mutation data. Data for mutation calls in primary and transplant tumor whole exome sequencing.
