# Supplementary material for: Single cell analysis reveals distinct immune landscapes in transplant and primary sarcomas that determine response or resistance to immunotherapy
Source: Nat Commun. 2020 Dec 17;11:6410. doi: 10.1038/s41467-020-19917-0 (PMC7746723; doi:10.1038/s41467-020-19917-0)
Supplement: Supplementary file 10 — Reporting Summary [file 41467_2020_19917_MOESM10_ESM.pdf]

nature research

Corresponding author(s): David G Kirsch, Yvonne M Mowery

Last updated by author(s): Oct 30, 2020

Reporting Summary

Nature Research wishes to improve the reproducibility of the work that we publish. This form provides structure for consistency and transparency in reporting. For further information on Nature Research policies, see our Editorial Policies and the Editorial Policy Checklist.

Statistics

For all statistical analyses, confirm that the following items are present in the figure legend, table legend, main text, or Methods section.

n/a	Confirmed	
☐	☒	The exact sample size (*n*) for each experimental group/condition, given as a discrete number and unit of measurement
☐	☒	A statement on whether measurements were taken from distinct samples or whether the same sample was measured repeatedly
☐	☒	The statistical test(s) used AND whether they are one- or two-sided<br>*Only common tests should be described solely by name; describe more complex techniques in the Methods section.*
☒	☐	A description of all covariates tested
☐	☒	A description of any assumptions or corrections, such as tests of normality and adjustment for multiple comparisons
☐	☒	A full description of the statistical parameters including central tendency (e.g. means) or other basic estimates (e.g. regression coefficient) AND variation (e.g. standard deviation) or associated estimates of uncertainty (e.g. confidence intervals)
☐	☒	For null hypothesis testing, the test statistic (e.g. *F*, *t*, *r*) with confidence intervals, effect sizes, degrees of freedom and *P* value noted<br>*Give P values as exact values whenever suitable.*
☒	☐	For Bayesian analysis, information on the choice of priors and Markov chain Monte Carlo settings
☒	☐	For hierarchical and complex designs, identification of the appropriate level for tests and full reporting of outcomes
☒	☐	Estimates of effect sizes (e.g. Cohen's *d*, Pearson's *r*), indicating how they were calculated

Our web collection on statistics for biologists contains articles on many of the points above.

Software and code

Policy information about availability of computer code

Data collection	No code was used for data collection
Data analysis	Bcl2Fastq2 conversion software provided by Illumina (v2.20.0.422), Base-calling RTA v3.3.3, FastQC (v0.11.5) and MultiQC (v1.0), Trimmomatic (v0.36), STAR (v2.5.4b), HTSeq66, DESeq2 (vl.20.0)67, gage (v2.34.0), CIBERSORTx (cibersortx.stanford.edu), ggplot2(3.3.0), ComplexHeatmap, Omicsoft Array Studio (vl0.0.1.118), BWA-MEM algorithm (v0.7.12), GATK (v3.8), GATK (v3.7) MuTect2, Ensembl YEP (Variant Effect Predictor, v88), netMHC (v4.0), samtools (v1.6), Fluidigm CyTOF software (v7.0), Cytobank, Cell Ranger Single Cell Software Suite (v2.l.1), Seurat R package (v2.4), Graphpad Prism (v8), (MSigDB v6.0), GSVA R package (vl.32.0), limma90 R package (v3.38.3), SingleR (vl.0.1), HALO image analysis software (v3.0.3). Computer codes used to generate survival curves, Fig. 4a, Supplementary Fig. 3a-c, and downstream scRNA-seq analysis in this manuscript can be found at https://gitlab.oit.duke.edu/wisdom2020/NatImmunol2020. The analysis for Fig. 2 and Supplementary Fig. 1 was performed with a proprietary pipeline and we are unable to publicly release this code. However, all raw data including those used to generate Fig. 2 and Supplementary Fig. 1 have been made publicly available and the implementation details in the Methods and Supplementary Information allow for independent replication of these results.

For manuscripts utilizing custom algorithms or software that are central to the research but not yet described in published literature, software must be made available to editors and reviewers. We strongly encourage code deposition in a community repository (e.g. GitHub). See the Nature Research guidelines for submitting code & software for further information.

Data

Policy information about availability of data

All manuscripts must include a data availability statement. This statement should provide the following information, where applicable:

- Accession codes, unique identifiers, or web links for publicly available datasets
- A list of figures that have associated raw data
- A description of any restrictions on data availability

All sequencing data have been deposited in publicly accessible databases: primary and transplant bulk tumor RNA-seq (NCBI Gene Expression Omnibus (GEO) database, accession number GSE148856); primary and transplant tumor whole exome sequencing (Bioproject database, project number PRJNA556574); scRNA-seq (SRA, accession number PRJNA556477); Rag2-/- and Rag2+/- bulk tumor RNA-seq (NCBI GEO database, accession number GSE154874); Rag2-/- and Rag2+/- tumor whole exome sequencing (Bioproject database, project number PRJNA630870). Mass cytometry data are available at flowrepository.org (ID FR-FCM-Z28C). All other relevant data are available from the corresponding authors upon reasonable request.

Field-specific reporting

Please select the one below that is the best fit for your research. If you are not sure, read the appropriate sections before making your selection.

☒ Life sciences ☐ Behavioural & social sciences ☐ Ecological, evolutionary & environmental sciences

For a reference copy of the document with all sections, see nature.com/documents/nr-reporting-summary-flat.pdf

Life sciences study design

All studies must disclose on these points even when the disclosure is negative.

Sample size	Sample sizes were based on those used in previous and preliminary studies from our lab. No statistical methods were used to predetermine sample size.
Data exclusions	One mass cytometry sample (mouse 522389) had a high fraction of dead cells (>75%), so this tumor was excluded from analysis and was not displayed in any figures. No other data were excluded.
Replication	All experiments presented in this study were performed using at least 2 biological replicates.
Randomization	Mice were randomly assigned to treatment groups for survival experiments, sequencing experiments, cytometric analysis, and histology.
Blinding	Investigators were blinded to mouse treatment groups during tumor measurement, tissue collection, histological analysis, and cytometric analysis.

Reporting for specific materials, systems and methods

We require information from authors about some types of materials, experimental systems and methods used in many studies. Here, indicate whether each material, system or method listed is relevant to your study. If you are not sure if a list item applies to your research, read the appropriate section before selecting a response.

Materials & experimental systems

n/a	Involved in the study	
☐	☒	Antibodies
☐	☒	Eukaryotic cell lines
☒	☐	Palaeontology and archaeology
☐	☒	Animals and other organisms
☒	☐	Human research participants
☒	☐	Clinical data
☒	☐	Dual use research of concern

Methods

n/a	Involved in the study	
☒	☐	ChIP-seq
☒	☐	Flow cytometry
☒	☐	MRI-based neuroimaging

Antibodies

Antibodies used	Antibodies used in this work are listed in Supplementary Tables 1 and 2.
Validation	Antibodies were validated by the manufacturers and information is available on their websites. Catalogue numbers are provided in Supplementary Tables 1 and 2.

Eukaryotic cell lines

Policy information about cell lines

Cell line source(s)	Cell lines used were generated in the Kirsch lab from autochthonous p53/MCA sarcomas.
Authentication	None of the cell lines used were authenticated.
Mycoplasma contamination	All cell lines tested negative for mycoplasma contamination.
Commonly misidentified lines (See ICLAC register)	N/A

Animals and other organisms

Policy information about studies involving animals; ARRIVE guidelines recommended for reporting animal research

Laboratory animals	The Trp53fl/fl allele used in this study has been described previously (Jonkers et al, Nature Genetics, 2001). Trp53fl/fl and wild type mice were maintained on a pure 129/SvJae genetic background and bred at Duke University. The Rag2-null allele used in this study has been described previously (Shinkai et al., Cell, 1992). Nude mice were purchased from Taconic. Additional information can be found in the methods section. Male and female mice and age-matched littermate controls were used for every experiment.
Wild animals	n/a
Field-collected samples	n/a
Ethics oversight	All animal studies were performed in accordance with protocols approved by the Duke University Institutional Animal Care and Use Committee (IACUC) and adhere to the NIH Guide for the Care and Use of Laboratory Animals.

Note that full information on the approval of the study protocol must also be provided in the manuscript.
